# Supplementary material for: Effectiveness and Safety of Ayurvedic Medicines in Type 2 Diabetes Mellitus Management: A Systematic Review and Meta-Analysis
Source: Front Pharmacol. 2022 Jun 8;13:821810. doi: 10.3389/fphar.2022.821810 (PMC9213670; doi:10.3389/fphar.2022.821810)
Supplement: Supplementary file 1 [file DataSheet1.zip › Supplementary Material/Forest and Funnel Plots/Tinospora cordifolia/FBG.pdf]

Tinospora cordifolia - FBG

Study	Intervention N	Intervention Mean	Intervention SD	Comparator N	Comparator Mean	Comparator SD	Mean Diff. with 95% CI	Weight (%)
Balasubramaniam 2010	20	135.00	14.00	20	137.00	19.00	-2.00 [-12.34, 8.34]	2.55
Mishra 2015	50	115.93	3.42	50	120.32	4.97	-4.39 [-6.06, -2.72]	97.45
**Overall**							-4.33 [-5.98, -2.68]	

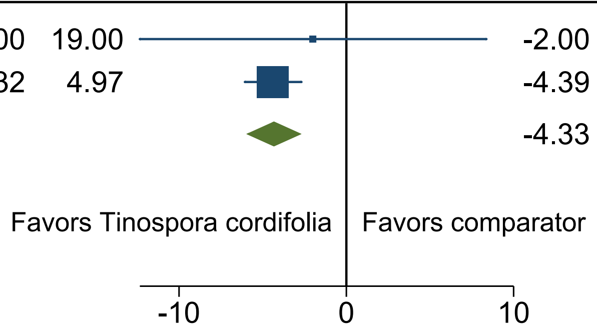

Heterogeneity: $\tau^2 = 0.00$, $I^2 = 0.00\%$, $H^2 = 1.00$

Test of $\theta_i = \theta_j$: Q(1) = 0.20, p = 0.65

Test of $\theta = 0$: z = -5.14, p = 0.00

Random-effects REML model
